# Complete Mitochondrial Genome and Its Phylogenetic Position in Red Algae *Fushitsunagia catenata* from South Korea

**DOI:** 10.3390/life14040534

**Published:** 2024-04-22

**Authors:** Maheshkumar Prakash Patil, Nur Indradewi Oktavitri, Young-Ryun Kim, Seokjin Yoon, In-Cheol Lee, Jong-Oh Kim, Kyunghoi Kim

**Affiliations:** 1 Industry-University Cooperation Foundation, Pukyong National University, 45 Yongso-ro, Nam-gu, Busan 48513, Republic of Korea; 2 Study Program of Environmental Engineering, Faculty of Science and Technology, Universitas Airlangga, Surabaya 60115, Indonesia; nur-i-o@fst.unair.ac.id; 3 Marine Eco-Technology Institute, Busan 48520, Republic of Korea; 4 Dokdo Fisheries Research Center, National Institute of Fisheries Science, Pohang 37709, Republic of Korea; 5 Department of Ocean Engineering, Pukyong National University, 45 Yongso-ro, Nam-gu, Busan 48513, Republic of Korea; 6 Department of Microbiology, Pukyong National University, 45 Yongso-ro, Nam-gu, Busan 48513, Republic of Korea; 7 School of Marine and Fisheries Life Science, Pukyong National University, 45 Yongso-ro, Nam-gu, Busan 48513, Republic of Korea

**Keywords:** *Fushitsunagia catenata*, mitogenome, phylogenetic analysis, red algae, Rhodymeniales

## Abstract

The mitogenome is an important tool in taxonomic and evolutionary studies. Only a few complete mitogenomes have been reported for red algae. Herein, we reported the complete mitochondrial genome sequence of *Fushitsunagia catenata* (Harvey) Filloramo, G.V. and Saunders, G.W. 2016, a monospecific genus. The genome was 25,889 bp in circumference and had a strongly biased AT of 70.4%. It consisted of 2 rRNAs, 23 tRNAs, and 24 protein-coding genes (PCGs). *nad5* (1986 bp) was the largest and *atp9* (231 bp) was the smallest PCG. All PCGs used ATG as an initiation codon and TAA as a termination codon, except TAG, which was the termination codon used in the *sdh3*, *rps3*, and *rps11* genes. The general structure and gene content of the present findings were almost identical to those of other red algae genomes, particularly those of the Rhodymeniales order. The maximum likelihood analysis showed that *F. catenata* was closely related to *Rhodymenia* pseudopalmata. The mitochondrial genome data presented in this study will enhance our understanding of evolution in Rhodophyta species.

## 1. Introduction

The phylum Rhodophyta, also known as red algae, is a monophyletic group largely comprising multicellular photosynthetic eukaryotes. The seven groups, Rhodophytes-Bangiophyceae, Compsopogonophyceae, Cyanidiophyceae, Florideophyceae, Porpyridiophyceae, Rhodellophyceae, and Stylonematophyceae, comprising over 7538 species, comprise a diverse group of algae [1]. The class Florideophyceae encompasses many species (7141), mostly multicellular sea algae. As eukaryotic members of the Archaeplastida supergroup, red algae are not real plants; they share similar ancestors with the green lineage (Chloroplastida) [2]. Red algae are uncommon in freshwater environments but ubiquitous in marine ecosystems (98%) [3]. 

Recent studies that combined morphological and genomic data resulted in numerous taxonomic revisions. The genus *Fushitsunagia* was recently isolated from the genus *Lomentaria* [4]. *Fushitsunagia catenata* is larger, measuring 10–15 cm in height, with straight apices, a turgid texture, and irregular branches [5]. The taxonomy of the genus *Fushitsunagia* is still unclear because morphological traits have limited taxonomic relevance. To date, only a few mitochondrial genes (*cob*, *cox1*, and *cox3*) of *F. catenata* have been reported. Phylogenetic analysis using complete mitochondrial genomes is more informative for determining evolutionary relatedness than single-gene sequencing [6]. Therefore, we analyzed the whole mitochondrial genome of *F. catenata* and discussed the evolutionary connections between rhodophytes.

*Fushitsunagia catenata* (Harvey) Filloramo, G.V. & Saunders, G.W. 2016 is a red macroalga and belongs to the phylum Rhodophyta (Florideophyceae; Rhodymeniophycidae; Rhodymeniales; Lomentariaceae) [7]. *Fushitsunagia* is a monospecific genus that is naturally found in China, Japan, and South Korea [8] and may be found in the Gulf of California [9], New South Wales, Australia [10], and Spain [11]. The cytochrome oxidase subunits and phylogenetic resolutions based on these genes have been reported [4]. 

Mitochondrial genes are valuable for phylogenetic research. However, a more precise understanding of phylogenetic relationships may be obtained by analyzing the full mitochondrial genome. There have been no reports of the full mitochondrial genome or phylogenetic analyses of *F. catenata*. This study included the construction of the first comprehensive mitochondrial genome of *F. catenata* using de novo assembly on an Illumina platform. The findings of this study will be important for future phylogenetic analysis, in-depth comprehension of gene content and structure, and comparative mitochondrial genome analyses.

## 2. Materials and Methods

### 2.1. Sample Collection and Genomic DNA Extraction

The red macroalga *F. catenata* sample (Figure S1) used in this study was collected from the coastal region of Gijang, Busan, South Korea (35.284634 N, 129.259071 E) in August 2022. The samples were subsequently deposited in the Ecological Restoration Group, Marine Eco-Technology Institute, Busan, South Korea (specimen number PU-T01-S-MA-05). Genomic DNA was isolated using a DNeasy Blood and Tissue kit (Qiagen, Germany) according to the manufacturer’s instructions. The concentration and purity of the extracted DNA were evaluated using a NanoDrop spectrophotometer (Thermo Fisher Scientific D1000, Waltham, MA, USA). The extracted genomic DNA was stored at a temperature of −4 °C and transported to Macrogen (Daejeon, South Korea; https://www.macrogen.com/ko/) for library creation and sequencing. 

### 2.2. Mitochondrial Genome Sequencing

DNA libraries were created using the TrueSeq Nano DNA Kit and then subjected to sequencing on the Illumina platform (Illumina, HiSeq 2500, San Diego, CA, USA) using paired-end reads with a length of 150 bp. To reduce analytical bias, the acquired reads were trimmed using the Trimmomatic v0.36 (http://www.usadellab.org/cms/?page=trimmomatic, accessed on 15 October 2023) [12]. This included the removal of adapter sequences and low-quality reads with quality scores below 20 (Q < 20). The trimmed reads were randomly sampled to assemble the mitochondrial genome. In this case, only the sampled reads were used for de novo assembly. The overall quality of sequencing reads was assessed using FastQC v0.11.5 (http://www.bioinformatics.babraham.ac.uk/projects/fastqc, accessed on 15 October 2023) [13]. High-quality reads were assembled using *k*-mers and SPAdes v3.15.0 (http://cab.spbu.ru/software/spades/, accessed on 15 October 2023) [14,15]. After the complete genome was assembled, BLAST analysis was performed to identify the contigs containing the mitogenome sequences in the NCBI database (https://blast.ncbi.nlm.nih.gov/Blast.cgi, accessed on 15 October 2023).

### 2.3. Mitochondrial Genome Assembly and Annotation

The contig was annotated using the online platform MFannot (https://megasun.bch.umontreal.ca/apps/mfannot/, accessed on 15 October 2023) [16]. Protein-coding genes (PCGs) were identified and validated using the open reading frame finder (https://www.ncbi.nlm.nih.gov/orffinder/, accessed on 15 October 2023) and verified manually using BLAST homology searches against the NCBI protein database [17]. The RNAweasel tool (https://megasun.bch.umontreal.ca/apps/rnaweasel/, accessed on 15 October 2023) was used to validate the annotated RNAs and detect introns [18]. The Tandem Repeats Finder tool (https://tandem.bu.edu/trf/, accessed on 15 October 2023) was used to detect and analyze repetitive sequences [19]. The assembled contig was subjected to identification analysis by querying BlastN and comparing its size with that of the previously reported mitochondrial genomes of Rhodophyta.

### 2.4. Physical Mapping and Codon Usage Analysis

Map visualization of the genetic information identified in the mitochondria of *F. catenata* (GenBank accession number OR045827) was generated using OGDRAW (https://chlorobox.mpimp-golm.mpg.de/OGDraw.html, accessed on 15 October 2023) [20]. The nucleotide content of the mitochondrial genome was determined using MEGA11 v11.0.8 software [21]. The codon usage of PCGs was analyzed using the Sequence Manipulation Suite program (https://www.bioinformatics.org/sms2/codon_usage.html, accessed on 15 October 2023) [22]. The skew analysis was determined using the following formulas: AT-skew = (A − T)/(A + T) and GC-skew = (G − C)/(G + C) [23]. Intergenic spacers between genes and overlapping areas were manually calculated.

### 2.5. Phylogenetic Analysis

The phylogenetic tree was constructed using the complete mitochondrial genome and the *cox1*, *cox3*, and *cob* gene sequences of 12 selected red algae from the subclass Rhodymeniophycidae, together with one outgroup member from the family Glaucocystaceae (Table 1). The mitochondrial genomes and gene sequences used in this study were retrieved from the NCBI GenBank database (https://www.ncbi.nlm.nih.gov/, accessed on 10 November 2023). Multiple sequence alignments were performed using ClustalW [24], and a maximum likelihood (ML) phylogenetic tree was created using MEGA11 [25]. ML analysis was conducted using the Tamura–Nei model with default settings and 1000 bootstrap replications [21].

## 3. Results

### 3.1. Mitochondrial Genome Characterization

**Figure 1:**
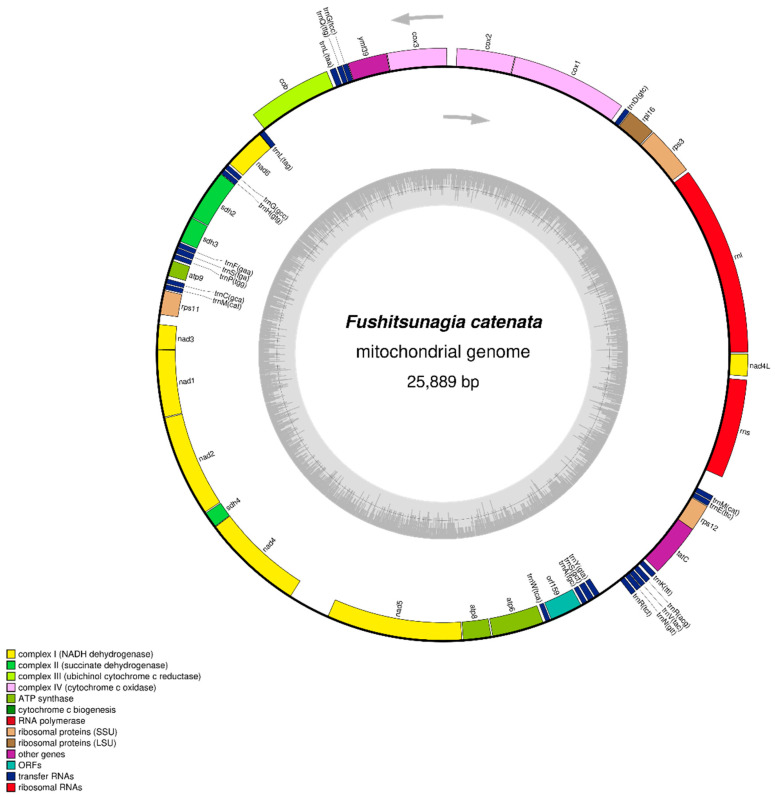
The circular mitochondrial genome of *Fushitsunagia catenata*. The arrow direction shows gene orientation, and the different colors reflect the groupings of functional genes together with their acronyms.

The *F. catenata* library was subjected to next-generation sequencing using an Illumina HiSeq 2500 sequencer, resulting in 22,066,120 raw reads. The GC content of the reads was 43.39%, with a Q20 score of 93.81% and a Q30 score of 87.08%. After removing low-quality sequences, 14,944,788 filtered reads were obtained with a GC content of 42.65%, Q20 accuracy of 98.86%, and Q30 accuracy of 95.78%. The reads were subjected to de novo assembly (100% coverage with a depth of 479.84), resulting in a contig consisting of 25,889 bases, with a GC content of 29.59%. The mitochondrial genome of *F. catenata* (GenBank: OR045827) was circular with a length of 25,889 bp (Figure 1). It contained 49 genes, consisting of 24 PCGs, 23 tRNAs, and 2 rRNAs. The H-strands contained 10 PCGs, 11 tRNAs, and 2 rRNAs. In contrast, the L strand consisted of 14 PCGs and 12 tRNAs. The nucleotide content of the whole genome was determined to be 37.1% A, 33.3% T, 15.1% G, and 14.5% C, as shown in Table 1. The analysis of nucleotide composition revealed a biased composition of A + T, which accounted for 70.4% of the total genome. The whole genome exhibited positive AT and GC skewness, suggesting a preference for using As over Ts and Gs over Cs.

### 3.2. Protein-Coding Genes

Twenty-four PCGs comprised 69.35% of the mitochondrial genome of *F. catenata*. These genes comprised a total length of 17,955 bp. There were clusters of genes such as NADH dehydrogenase subunits, succinate dehydrogenase, apocytochrome b, cytochrome c oxidase, ATP synthase, small- and large-subunit ribosomal proteins, independent protein translocase, and a gene encoding a hypothetical protein (Table 2). The *nad5* and *atp9* genes were the largest and smallest, respectively, in terms of length within the whole mitochondrial genome. *Nad5* accounted for 7.67% (1986 bp) of the genome, whereas *atp9* accounted for 0.89% (231 bp). ATG and TAA served as the initiation and termination codons for all PCGs, except TAG, which served as the termination codon specifically for the *sdh3*, *rps3*, and *rps11* genes. The mitochondrial genome of *F. catenata* contained only *rpl16* among the ribosomal protein genes (Table 3). The genes *rpl5* and *rpl20* do not exist, and no intronic coding sequences were identified.

### 3.3. Codon Usage Analysis

A codon usage analysis of the mitochondrial genome of *F. catenata* showed that 5961 amino acids were expressed in the PCGs (Table S1). The amino acid composition indicated that leucine (N = 900, 15.10%), phenylalanine (N = 587, 9.85%), and isoleucine (N = 582, 9.76%) were the amino acids most often found. Conversely, cysteine (N = 78, 1.31%), histidine (N = 121, 2.03%), and tryptophan (N = 129, 2.16%) showed the lowest levels of abundance among the identified amino acids. The codons TTA (leucine, N = 568, 9.53%), TTT (phenylamine, N = 515, 8.64%), and ATT (alanine, N = 388, 6.51%) were the most frequently used codons in PCGs.

### 3.4. RNAs

In the mitochondrial genome of *F. catenata*, the rRNA genes were located on the H-strand and identified as *rnl* (large subunit, 2604 bp) and *rns* (small subunit, 1361 bp) (Table 2). These genes had a combined length of 3965 bp, accounting for 15.32% of the whole mitochondrial genome. The rRNA genes were separated using the *nad4L* gene. A total of 23 tRNAs, ranging from 71 to 93 bp in length, were found in the mitochondrial genome; trnI was not identified in the *F. catenata* mitochondrial genome. Among these, arginine (*trnR*-TCT and *trnR*-ACG), glycine (*trnG*-TCC and *trnG*-GCC), leucine (*trnL*-TAA and trnL-TAG), methionine (*trnM*-CAT), and serine (*trnS*-TGA and trnS-GCT) had two copies with distinct anticodons, with the exception of methionine, which had the same anticodon. The tRNA cysteine (*trnC*-GCA, 71 bp) was the shortest and serine (*trnS*-GCT, 93 bp) was the longest. The total tRNA length was 1741 bp, accounting for 6.73% of the whole genome length, and no intronic RNA sequences were detected.

### 3.5. Overlapping and Intergenic Spacer Regions

An examination of the intergenic nucleotides of the *F. catenata* mitochondrial genome sequence revealed that only two gene junctions exhibited an overlap of 21 bp: *trnL*–*nad6* (1 bp overlap) and *TatC*–*rps12* (20 bp overlap). In addition, we observed intergenic gaps ranging from 1 to 607 bp. The largest intergenic gap, measuring 607 bp, was observed between *nad4* and *nad5* genes (Table 2).

### 3.6. Phylogenetic Analysis

**Figure 2:**
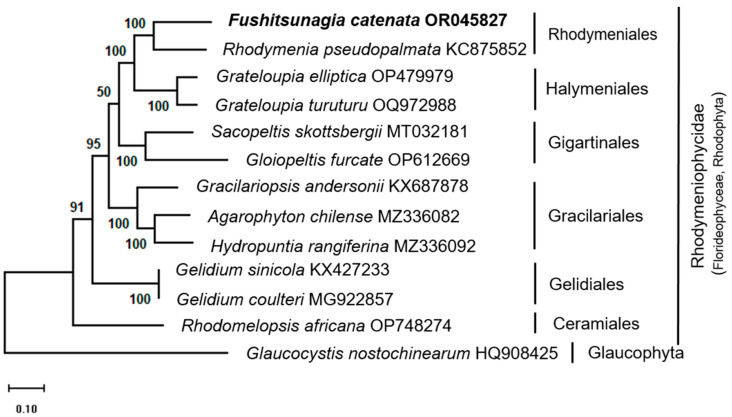
Maximum likelihood phylogenetic tree based on complete mitochondrial genome sequence of Rhodymeniophycidae. The sequence generated in this study is in bold. The support value on each node represents the bootstrap value.

ML phylogenetic trees were constructed with complete mitochondrial genome sequences based on single-gene sequences of the species within the Rhodymeniales order. The ML phylogenetic analysis indicated that *F. catenata* was most closely related to *Rhodymenia pseudopalmata* with strong bootstrap support (Figure 2). Rhodymeniales species (*F. catenata* and *R. pseudopalmata*) formed a monophyletic clade with Halymeniales species (*Grateloupia elliptica*, *G. turuturu*) with high bootstrap support but not with other species.

A phylogenetic analysis, using gene sequences of *cox1* (Figure S2), *cox3* (Figure S3), and *cob* (Figure S4), revealed differences in the relationships among the species in the group. However, the bootstrap values supporting each node were often modest, except for a substantial bootstrap value that supported the relationship between sister taxa of *Grateloupia* and *Gelidium* species. Most algal orders formed a monophyletic group in the phylogenies of *cox1*, *cox3*, and *cob*, except for Gigartinales and Rhodymeniales in the *cox1* phylogeny.

## 4. Discussion

The mitochondrial genome of *F. catenata* conformed to characteristics often observed in red algae, and the quality of the sequenced genome was comparable to that of other species belonging to the Rhodymeniophycidae subfamily (Table 1). The size and base composition of monospecific *F. catenata* were consistent with those of a previously reported Rhodymeniales species, *R. pseudopalmata* (KC875852; 26,166 bp, 29.5% GC) [29]. 

The long intergenic nucleotide region between *nad4* and *nad5* in *F. catenata* (Table 2) was similar to the intergenic regions of the previously reported species, *G. elliptica*, *Gelidium coulteri*, *G. sinicola*, *Gracilariopsis andersonii*, *G. turuturu*, and *Sarcopeltis skottsbergii*. In contrast, the other species investigated in this study possessed intronic tRNA genes between *nad4* and *nad5* (specifically, *trnI* in *Agarophyton chilense*, *Hydropuntia rangiferina*, *Rhodomelopsis africana*, *R. pseudopalmata*, and *trnH* in *Gloiopeltis furcate*) [6,26,27,28,29,30]. The lack of *trnI* in some species may be attributed to the inaccurate annotation of tRNA. The most closely related species, *R. pseudopalmata* (Figure S5, circular mitogenome), had gene content similar to that of *F. catenata* [12]. Three tRNAs (*trnH*, *trnW*, and *trnY*) were present in *R. pseudopalmata* but absent in *F. catenata*, whereas two tRNAs (*trnI* and *trnU*) were absent in *F. catenata* but present in *R. pseudopalmata*. Additionally, we found that both species had two copies of each *trnL*, *trnG*, *trnS*, and *trnM* gene and that *F. catenata* alone had two copies of the *trnR* gene.

In general, the arrangement of mitochondrial genomes in Rhodymeniophycidae is highly conserved in terms of genome size and gene content (Table 3), as often observed in other red algal groups [6,26,29]. No intronic PCGs or tRNA were detected in the mitochondrial genome of *F. catenata*. However, group II intronic *cox1* genes have been identified in red algae, such as *G. elliptica* and *G. turuturu*, as reported by Patil et al. [27,28]. Additionally, intronic tRNA genes have been reported, including the intronic *trnI* gene in *A. chilense* (MZ336082), *H. rangiferina* (MZ336092), *R. africana* (OP748274), and *R. pseudopalmata* (KC875852), and the intronic *trnH* gene in *G. furcate* (OP612669). Most red algal species that have been sequenced have two rRNAs, *rnl* and *rns*. However, two species of the Halymeniales order have an additional rRNA called *rns5* [27,28]. rRNA has also been detected in other Florideophyceae red algal species. However, they only exist in certain orders and species of the same genus or family [6,31]. Among the ribosomal protein genes, the *rpl20* gene seems to be the least conserved in red algae [6]. *rpl5* and *rpl20* were not identified in the mitochondrial genome sequence of *F. catenata*, which is consistent with the mitogenome characteristics of *G. coultery* (MG922857), *G. sinicola* (KX427233), *R. africana* (OP748274), and *R. pseudopalmata* (KC875852). These differences may play important roles in the mitogenomic evolution of Florideophyceae red algae. 

The ML phylogenetic analysis, based on the complete genome sequence (Figure 2) of selective members of Rhodymeniophycidae, revealed that *F. catenata* is closely related to *R. pseudopalmata*, which belongs to the Rhodymeniales order. Furthermore, *F. catenata* formed a monophyletic group with species from the Halymeniales order, namely *G. elliptica* (OP479979) and *G. turuturu* (OQ972988). A previous study documented the emergence of a monophyletic cluster, including the Rhodymeniales and Halymeniales orders [31]. However, the phylogenetic tree based on individual genes (Figures S2–S4) shows that the *cob* and *cox3* genes have a phylogenetic structure similar to that of the complete genome-based phylogeny. However, the phylogeny based on the *cox1* gene sequence separated the sister species of the monophyletic clade of Gigartinales (*G. furcate*, *S. skottsbergii*) and Rhodymeniales (*F. catenata*, *R. pseudopalmata*). The resulting topology was aligned with a phylogenetic tree constructed from multiple genes and the complete mitochondrial genome sequence [27,28,31]. Filloramo and Sanders [4] found that the Rhodymeniales order is monophyletic and divided into two major lineages: Fryeellaceae, which is a sister of Faucheaceae and Lomentariaceae, and Rhodymeniaceae, which is allied to Champiaceae and Hymenocladiaceae. However, complete mitochondrial genome sequences of several Rhodymeniales families are unavailable. Therefore, to understand the differences and relationships between species, an extensive phylogenetic study of the whole mitochondrial genome of Rhodymeniales is necessary.

## 5. Conclusions

The present study investigated the complete mitochondrial genome of the red alga *F. catenata* (NCBI GenBank accession no. OR045827.1) and analyzed their genomic and phylogenetic relationships with other species. In this study, we observed that the *F. catenata* mitochondrial genome has lost ribosomal protein genes (*rpl5* and *rpl20*), in contrast with other red algae. Furthermore, the *trnI* gene was not identified, which may have been due to annotation errors. This study demonstrated that the *F. catenata* mitochondrial genome exhibits characteristics common to red algae and that the quality of its sequenced mitochondrial genome is similar to that of other species within the Rhodymeniophycidae subfamily. The phylogenies presented in this study, which were based on the mitochondrial genome, indicate that it is not possible to accurately determine species relationships within algal orders by analyzing individual gene sequences, such as *cox1*, *cox3*, *cob*, and entire genome sequences. Therefore, it is necessary to explore multi-gene approaches. It is expected that the mitochondrial genome data reported in this study will be valuable for enhancing our understanding of evolution in Rhodophyta species.

## Figures and Tables

**Table 1:** List of algal mitochondrial genomes with nucleotide compositions.

Algae Name	Accession Number	Length (bp)	Nucleotide Composition (%)						AT-Skew	GC-Skew	Ref.
			A	T	G	C	AT	GC			
*Agarophyton chilense*	MZ336082	25,942	37.9	34.6	14.0	13.4	72.5	27.4	0.0455	0.0219	-
*Fushitsunagia catenata*	OR045827	25,889	37.1	33.3	15.1	14.5	70.4	29.6	0.0540	0.0203	This study
*Gelidium coulteri*	MG922857	24,963	36.8	33.0	15.3	14.9	69.8	30.2	0.0544	0.0132	-
*Gelidium sinicola*	KX427233	24,969	36.8	33.0	15.3	14.9	69.8	30.2	0.0544	0.0132	[26]
*Gloiopeltis furcate*	OP612669	26,600	32.8	29.2	19.3	18.7	62.0	38.0	0.0581	0.0158	-
*Gracilariopsis andersonii*	KX687878	27,011	37.5	34.5	14.4	13.6	72.0	28.0	0.0417	0.0286	[6]
*Grateloupia elliptica*	OP479979	28,503	36.2	32.6	15.9	15.3	68.8	31.2	0.0523	0.0192	[27]
*Grateloupia turuturu*	OQ972988	28,265	36.1	32.7	16.1	15.1	68.8	31.2	0.0494	0.0321	[28]
*Hydropuntia rangiferina*	MZ336092	25,908	39.1	35.5	12.9	12.5	74.6	25.4	0.0483	0.0157	-
*Rhodomelopsis africana*	OP748274	26,394	39.7	35.8	12.5	12.0	75.5	24.5	0.0517	0.0204	-
*Rhodymenia pseudopalmata*	KC875852	26,166	36.9	33.6	15.0	14.5	70.5	29.5	0.0468	0.0169	[29]
*Sarcopeltis skottsbergii*	MT032181	25,908	37.5	34.0	14.6	13.9	71.5	28.5	0.0490	0.0246	[30]
*Glaucocystis nostochinearum*	HQ908425	34,087	38.4	35.9	13.0	12.7	74.3	25.7	0.0336	0.0117	-

**Table 2:** *Fushitsunagia catenata* (OR045827) mitochondrial gene annotation.

Gene Group		Gene	Three Letter Code	Strand	Location		Size (bp)	No. of Amino Acids	Start Codon	Stop Codon	Anticodon	Intergenic Nucleotides *
					Start	End						
rRNA	Large subunit of a ribosome	*rnl*	-	H	20	2623	2604	-	-	-	-	36
	Small subunit of a ribosome	*rns*	-	H	24,176	25,536	1361	-	-	-	-	47
tRNA	Transfer RNA genes	*trnD*	Asp	H	3765	3836	72	-	-	-	GTC	55
		*trnG*	Gly	H	7789	7860	72	-	-	-	TCC	7
		*trnQ*	Gln	H	7868	7939	72	-	-	-	TTG	19
		*trnL*	Leu	H	7959	8043	85	-	-	-	TAA	42
		*trnL*	Leu	L	9266	9348	83	-	-	-	TAG	−1
		*trnG*	Gly	L	9981	10,052	72	-	-	-	GCC	15
		*trnH*	His	L	10,068	10,142	75	-	-	-	GTG	3
		*trnF*	Phe	L	11,302	11,375	74	-	-	-	GAA	8
		*trnS*	Ser	L	11,384	11,468	85	-	-	-	TAG	20
		*trnP*	Pro	L	11,482	11,554	73	-	-	-	TGG	26
		*trnC*	Cys	L	11,854	11,924	71	-	-	-	GCA	12
		*trnM*	Met	L	11,937	12,010	74	-	-	-	CAT	15
		*trnW*	Trp	L	20,928	20,999	72	-	-	-	TCA	13
		*trnA*	Ala	L	21,517	21,590	74	-	-	-	TGC	21
		*trnS*	Ser	L	21,612	21,704	93	-	-	-	GCT	19
		*trnY*	Tyr	L	21,724	21,804	81	-	-	-	GTA	351
		*trnR*	Arg	H	22,156	22,230	75	-	-	-	TCT	17
		*trnN*	Asn	H	22,248	22,321	74	-	-	-	GTT	5
		*trnV*	Val	H	22,327	22,398	72	-	-	-	TAC	14
		*trnR*	Arg	H	22,413	22,486	74	-	-	-	ACG	33
		*trnK*	Lys	H	22,520	22,593	74	-	-	-	TTT	35
		*trnE*	Glu	H	23,744	23,815	72	-	-	-	TTC	8
		*trnM*	Met	H	23,824	23,896	73	-	-	-	CAT	279
PCGs	NADH dehydrogenase subunits (complex 1)	*nad6*	-	L	9348	9956	609	202	ATG	TAA	-	24
		*nad3*	-	L	12,519	12,884	366	121	ATG	TAA	-	1
		*nad1*	-	L	12,886	13,866	981	326	ATG	TAA	-	8
		*nad2*	-	L	13,875	15,359	1485	494	ATG	TAA	-	12
		*nad4*	-	L	15,615	17,096	1482	493	ATG	TAA	-	607
		*nad5*	-	L	17,704	19,689	1986	661	ATG	TAA	-	14
		*nad4L*	-	H	25,584	25,889	306	101	ATG	TAA	-	19
	Succinate dehydrogenase (complex 2)	*sdh2*	-	L	10,146	10,895	750	249	ATG	TAA	-	9
		*sdh3*	-	L	10,905	11,285	381	126	ATG	TAG	-	16
		*sdh4*	-	L	15,372	15,611	240	79	ATG	TAA	-	3
	Apocytochrome b (complex 3)	*cob*	-	H	8086	9246	1161	386	ATG	TAA	-	19
	Cytochrome c oxidase (complex 4)	*cox1*	-	H	3892	5487	1596	531	ATG	TAA	-	4
		*cox2*	-	H	5492	6289	798	265	ATG	TAA	-	131
		*cox3*	-	H	6421	7239	819	272	ATG	TAA	-	3
	ATP synthase (complex 5)	*ymf39*	-	H	7243	7785	543	180	ATG	TAA	-	3
		*atp9*	-	L	11,581	11,811	231	76	ATG	TAA	-	42
		*atp8*	-	L	19,704	20,114	411	136	ATG	TAA	-	18
		*atp6*	-	L	20,133	20,891	759	252	ATG	TAA	-	36
	SSU ribosomal protein	*rps3*	-	H	2660	3340	681	226	ATG	TAG	-	12
		*rps11*	-	L	12,026	12,379	354	117	ATG	TAG	-	139
		*rps12*	-	H	23,363	23,737	375	124	ATG	TAA	-	6
	LSU ribosomal protein	*rpl16*	-	H	3353	3757	405	134	ATG	TAA	-	7
	Independent protein translocase	*TatC*	-	H	22,629	23,384	756	251	ATG	TAA	-	−20
	Hypothetical proteins	*orf159*	-	L	21,013	21,492	480	159	ATG	TAA	-	24

Note: H and L indicate that the genes were transcribed on the heavy and light strands, respectively; * denotes the number of nucleotides between a given gene and the next, with a negative value indicating an overlap.

**Table 3:** *Fushitsunagia catenata* and reference algal mitochondrial genome features.

Algae Name	RNA			Protein-Coding Genes (PCGs)											Intronic PCG/tRNA
	Total tRNA	rRNA		Total PCG	*atp4*, *atp6*, *atp8*, *atp9*	*cob*, *cox1*, *cox2*, *cox3*	*nad1*, *nad2*, *nad3*, *nad4*, *nad4L*, *nad5*, *nad6*	*Ribosomal proteins*				*sdh2*, *sdh3*, *sdh4*	*tatC*	No. of ORF*	
		*rnl*, *rns*	*rrn5*					*rpl5*	*rpl16*	*rpl20*	*rps3*, *rps11*, *rps12*				
*A. chilense*	23	+	-	25	+	+	+	-	+	+	+	+	+	1	*trnI*
*F. catenata*	23	+	-	24	+	+	+	-	+	-	+	+	+	1	-
*G. coulteri*	20	+	-	23	+	+	+	-	+	-	+	+	+	-	-
*G. sinicola*	18	+	-	23	+	+	+	-	+	-	+	+	+	-	-
*G. furcata*	23	+	-	24	+	+	+	-	+	+	+	+	+	-	*trnH*
*G. andersonii*	18	+	-	25	+	+	+	-	+	+	+	+	+	1	-
*G. elliptica*	20	+	+	26	+	+	+	+	+	+	+	+	+	1	*cox1*
*G. turuturu*	20	+	+	26	+	+	+	-	+	+	+	+	+	2	*cox1*
*H. rangiferina*	24	+	-	25	+	+	+	-	+	+	+	+	+	1	*trnI*
*R. africana*	22	+	-	23	+	+	+	-	+	-	+	+	+	-	*trnI*
*R. pseudopalmata* ^a^	21	+	-	24	+	+	+	-	+	-	+	+	+	1	*trnI*
*S. skottsbergii*	22	+	-	24	+	+	+	-	+	+	+	+	+	-	-
*G. nostochinearum* ^a,b^	25	+	+	34	+	+	+	+	+	-	+	*sdh2*-	-	4	-

Note: ‘+’ indicates present and ‘-’ indicates absent. ORF* represents the hypothetical proteins. ‘^a^’ indicates PCGs *atp4*/*ymf39* is annotated as a hypothetical protein. ‘^b^’ indicates additional NADH dehydrogenase subunits (*nad7*, *nad9*, and *nad11*) and additional PCG including rpl (*rpl2*, *rpl6*) and rps (*rps7*, *rps10*, *rps13*, and *rps19*).

## Data Availability

The mitogenome sequence data supporting the findings of this study are openly available in GenBank at https://www.ncbi.nlm.nih.gov/ under the accession number OR045827. The associated BioProject, BioSample, and SRA numbers are PRJNA1046667, SAMN38505473, and SRR26992496, respectively.

## Supplementary Materials

The following supporting information can be downloaded at: https://www.mdpi.com/article/10.3390/life14040534/s1, Figure S1. A specimen image of *Fushitsunagia catenata*, a macroalga that was collected from the East Sea in South Korea. It is about 11 to 14 cm tall, has straight and hard apices, and irregular branching. Figure S2. The phylogenetic tree generated from maximum likelihood (ML) analysis for the cox1 gene sequences of several algae species. The sequence generated in this study is in bold. Numbers at nodes represent the bootstrap values based on 1000 replicates. Figure S3. The phylogenetic tree generated from maximum likelihood (ML) analysis for the cox3 gene sequences of several algae species. The sequence generated in this study is in bold. Numbers at nodes represent the bootstrap values based on 1000 replicates. Figure S4. The phylogenetic tree generated from maximum likelihood (ML) analysis for the cob gene sequences of several algae species. The sequence generated in this study is in bold. Numbers at nodes represent the bootstrap values based on 1000 replicates. Figure S5. The circular mitochondrial genome of Rhodymenia pseudopalmata (GenBank accession no. KC875852). Map visualization was produced using OGDRAW. The colors reflect the grouping of functional genes together with their acronyms. Table S1. Codon usage of *Fushitsunagia catenata* (OR045827) mitochondrial protein-coding genes.
