# Supplementary material for: Complete Mitochondrial Genome and Its Phylogenetic Position in Red Algae Fushitsunagia catenata from South Korea
Source: Life (Basel). 2024 Apr 22;14(4):534. doi: 10.3390/life14040534 (PMC11050817; doi:10.3390/life14040534)
Supplement: Supplementary file 1 [file life-14-00534-s001.zip › life-2902743-supplementary.pdf]

Supplementary Data

Complete Mitochondrial Genome and Its Phylogenetic Position in Red Algae *Fushitsunagia catenata* from South Korea

Maheshkumar Prakash Patil [1], Nur Indradewi Oktavitri [2], Young-Ryun Kim [3], Seokjin Yoon [4], In-Cheol Lee [5], Jong-Oh Kim [6,7,*] and Kyunghoi Kim [2,5,*]

[1] Industry-University Cooperation Foundation, Pukyong National University, 45 Yongso-ro, Nam-gu, Busan 48513, Republic of Korea
[2] Study Program of Environmental Engineering, Faculty of Science and Technology, Universitas Airlangga, Surabaya 60115, Indonesia; nur-i-o@fst.unair.ac.id
[3] Marine Eco-Technology Institute, Busan 48520, Republic of Korea
[4] Dokdo Fisheries Research Center, National Institute of Fisheries Science, Pohang 37709, Republic of Korea
[5] Department of Ocean Engineering, Pukyong National University, 45 Yongso-ro, Nam-gu, Busan 48513, Republic of Korea
[6] Department of Microbiology, Pukyong National University, 45 Yongso-ro, Nam-gu, Busan 48513, Republic of Korea
[7] School of Marine and Fisheries Life Science, Pukyong National University, 45 Yongso-ro, Nam-gu, Busan 48513, Republic of Korea
* Correspondence: jokim@pknu.ac.kr (J.-O.K.); hoikim@pknu.ac.kr (K.K.); Tel.: +82-51-629-6583 (K.K.); Fax: +82-51-629-6590 (K.K.)

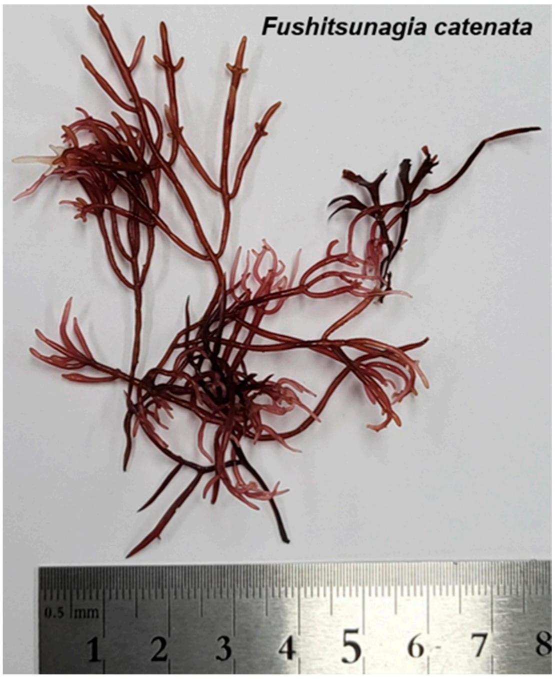

Figure S1. A specimen image of *Fushitsunagia catenata*, a macroalga that was collected from the East Sea in South Korea. It is about 11 to 14 cm tall, has straight and hard apices, and irregular branching.

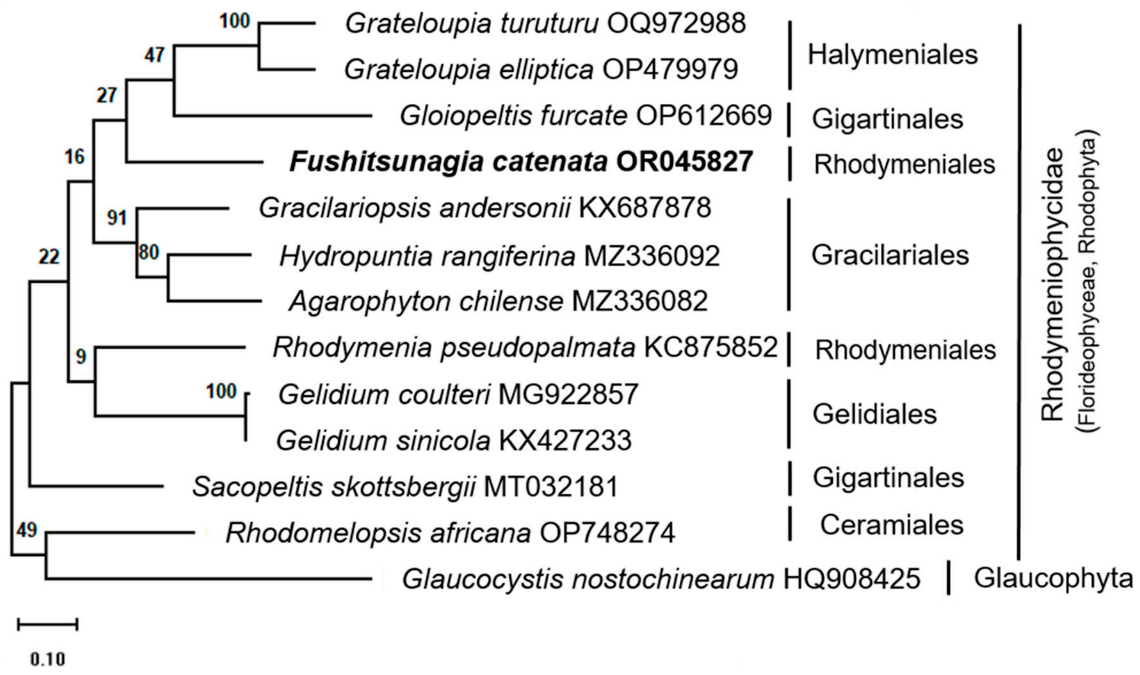

Figure S2. The phylogenetic tree generated from maximum likelihood (ML) analysis for the *cox1* gene sequences of several algae species. The sequence generated in this study is in bold. Numbers at nodes represent the bootstrap values based on 1000 replicates.

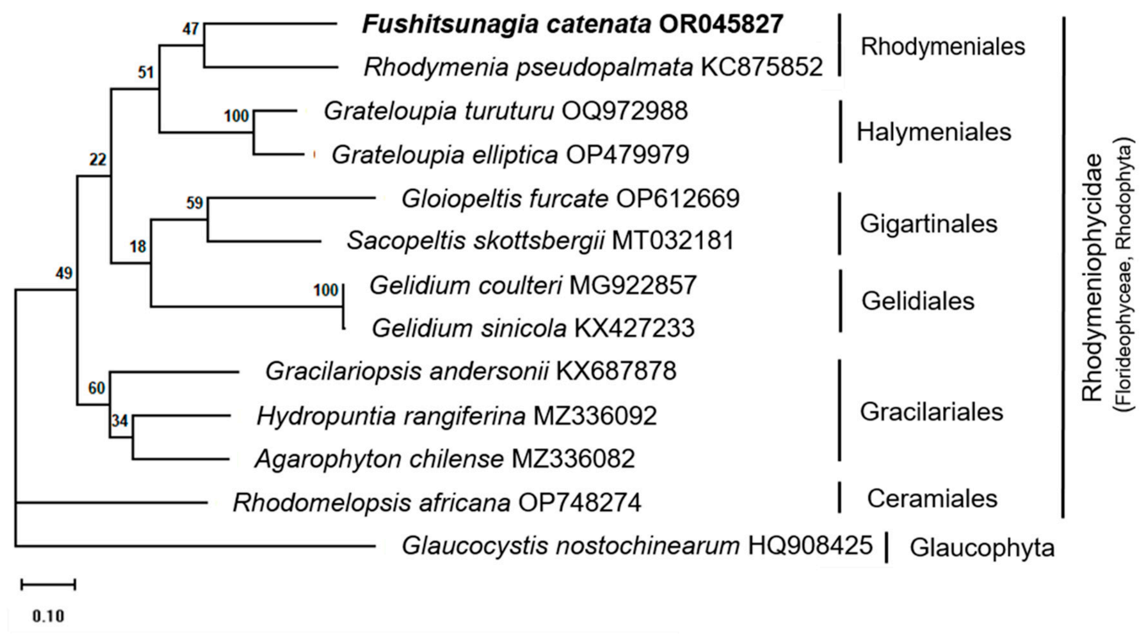

Figure S3. The phylogenetic tree generated from maximum likelihood (ML) analysis for the *cox3* gene sequences of several algae species. The sequence generated in this study is in bold. Numbers at nodes represent the bootstrap values based on 1000 replicates.

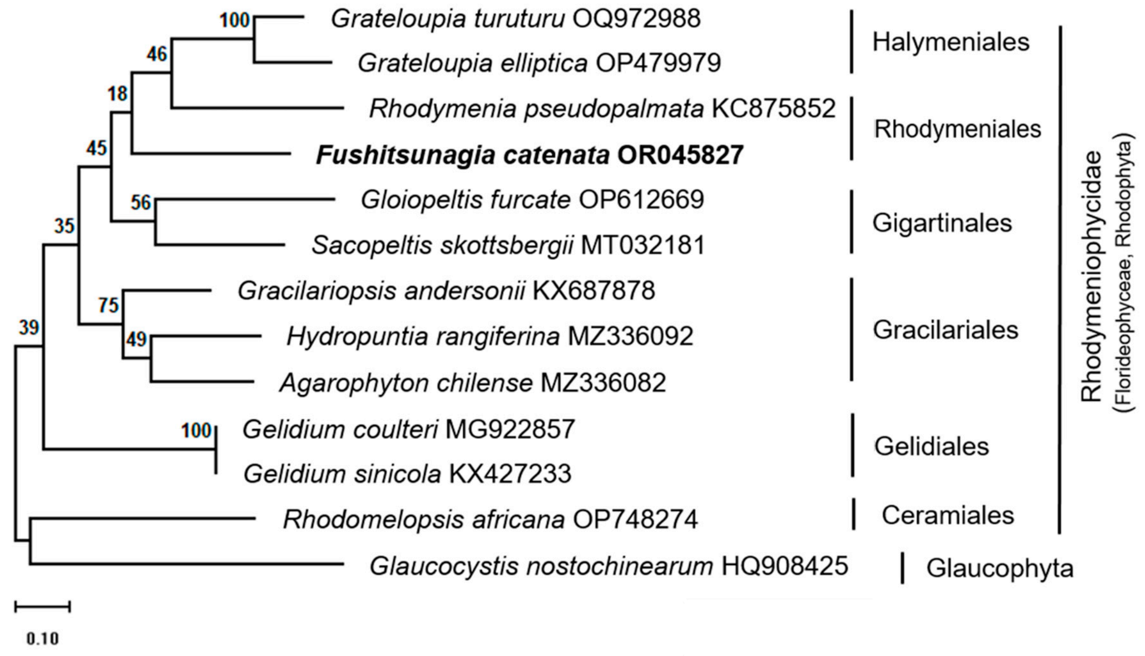

Figure S4. The phylogenetic tree generated from maximum likelihood (ML) analysis for the *cob* gene sequences of several algae species. The sequence generated in this study is in bold. Numbers at nodes represent the bootstrap values based on 1000 replicates.

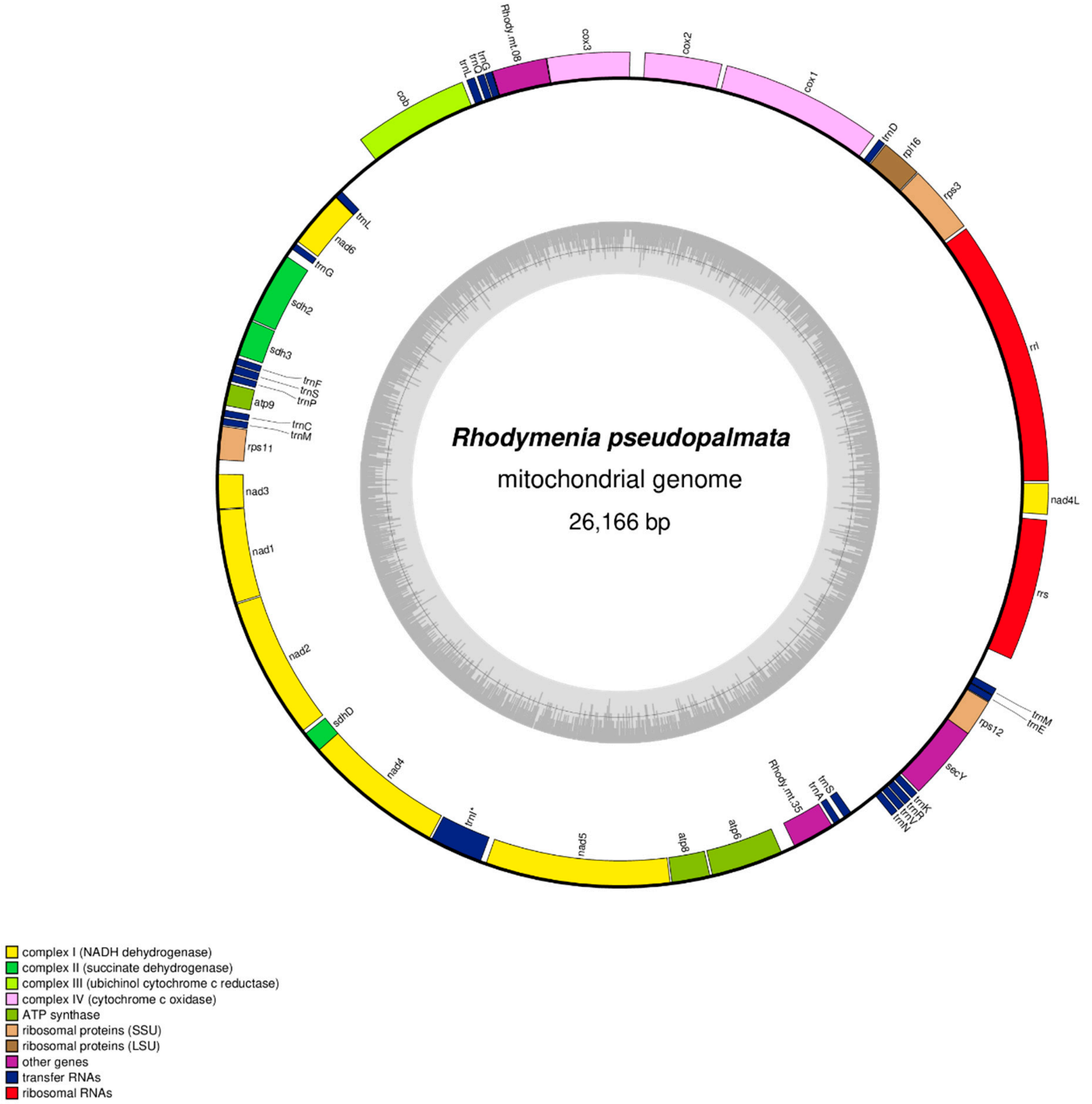

Figure S5. The circular mitochondrial genome of *Rhodymenia pseudopalmata* (GenBank accession no. KC875852). Map visualization was produced using OGDRAW. The colors reflect the grouping of functional genes together with their acronyms.

Table S1. Codon usage of *Fushitsunagia catenata* (OR045827) mitochondrial protein-coding genes.

AA	Codon	No.	%	Fraction	AA	Codon	No.	%	Fraction	AA	Codon	No.	%	Fraction
Ala	GCG	27	0.453	0.09	Lys	AAG	32	0.537	0.13	Arg	CGT	42	0.705	0.31
	GCA	125	2.097	0.41		AAA	224	3.758	0.88		CGC	17	0.285	0.13
	GCT	132	2.214	0.43	Leu	TTG	118	1.980	0.13	Ser	AGT	107	1.795	0.21
	GCC	23	0.386	0.07		TTA	568	9.529	0.63		AGC	35	0.587	0.07
Cys	TGT	54	0.906	0.69		CTG	16	0.268	0.02		TCG	32	0.537	0.06
	TGC	24	0.403	0.31		CTA	75	1.258	0.08		TCA	174	2.919	0.34
Asp	GAT	117	1.963	0.87		CTT	108	1.812	0.12		TCT	137	2.332	0.27
	GAC	17	0.285	0.13		CTC	15	0.252	0.02		TCC	26	0.436	0.05
Glu	GAG	31	0.554	0.21	Met	ATG	157	2.634	1.0	Thr	ACG	27	0.453	0.08
	GAA	122	2.047	0.79	Asn	AAT	208	3.489	0.76		ACA	109	1.829	0.34
Phe	TTT	515	8.639	0.88		AAC	66	1.107	0.24		ACT	151	2.533	0.47
	TTC	72	1.208	0.12	Pro	CCG	16	0.268	0.08		ACC	33	0.554	0.10
Gly	GGG	41	0.688	0.13		CCA	75	1.258	0.38	Val	GTG	32	0.537	0.08
	GGA	100	1.678	0.31		CCT	88	1.476	0.44		GTA	139	2.332	0.36
	GGT	140	2.349	0.43		CCC	19	0.319	0.10		GTT	191	3.204	0.50
	GGC	43	0.721	0.13	Gln	CAG	25	0.419	0.15		GTC	23	0.386	0.06
His	CAT	96	1.610	0.79		CAA	138	2.315	0.85	Trp	TGG	46	0.772	0.36
	CAC	25	0.419	0.21	Arg	AGG	14	0.235	0.10		TGA	83	1.392	0.64
Ile	ATA	139	2.332	0.24		AGA	37	0.621	0.27	Tyr	TAT	168	2.818	0.69
	ATT	388	6.509	0.67		CGG	8	0.134	0.06		TAC	74	1.241	0.31
	ATC	55	0.923	0.09		CGA	18	0.302	0.13					
